# How Co-Operation Must Begin

**Published:** 1921-07-30

**Authors:** 


### HOW CO-OPERATION MUST BEGIN.

Though there is a strong desire for co-operation to help in the solution of hospital difficulties, the recent discussions in the House of Lords and at the luncheon at the Royal Automobile Club lacked definite proposals, except in so far as they urged the Government to pass a short Bill to enable Boards of Guardians to co-operate without fear of transgressing the letter of the law. In this state of affairs a simple suggestion may be timely, especially as a point is given to it by the procedure to be adopted in the distribution of the Government grant. It is agreed that no mechanical method of assessment should be adopted, and that speedy help is wanted for those hospitals which are most in need at the moment. But to discuss those hospitals which are most in need is, for want of a simple reform, much less easy than it sounds. How is the Commission to ascertain which hospitals are most in need when the financial year is not the same for all of them? We have, indeed, a Uniform System of accounts, generally, if not universally, adopted (and here we may remark that even some of the largest provincial hospitals only partially adopt this system). But even if its adoption were universal the uniformity is often misleading, because the financial year is not the same for all. Let us admit at once that you cannot have a real uniform system of accounts unless all hospitals close their financial year upon the same date. Illustration of this fact is hardly necessary, but one may be provided to show the present defect. In a certain town a special effort was made by the working classes for the benefit of their local hospitals, and the money was divided at the end of the year. But one of the hospitals closes its accounts two or three months before the others, and, therefore, the sum received is not included in its accounts for the year. The result is that it shows a loss or deficit, and may seem to be in greater need than it really is. Consequently, any grant of urgency made to this hospital will be based on information unsatisfactory in itself, and possibly unfair to other neighbouring hospitals.

Now, with so small a sum to be distributed, the grants in any event are likely to cause heart-searchings and disappointment, but the excuse for these could be much reduced if the Regional Committees of the British Hospitals Association and the new committees now being formed insisted as a preliminary that the same financial year should be adopted by all hospitals in their area. It does not so much matter what the dates are so long as they are identical, but, just as the Uniform System is general for all areas, so the financial year should preferably be the same throughout the country. This preliminary reform is not difficult, is important, and can be introduced without delay. The distribution of the grant provides a strong motive for its adoption, since, without it, the local committees have not the information which they need to assess fairly the urgency of different hospitals' requirements.

Any plan of co-ordination must be ineffective until this preliminary step has been taken. It is a simple and practical step on the road, which can be adopted without raising the difficult questions which make amalgamation schemes and the like so thorny. No hospital's independence would suffer by it, and without it co-ordination is not really practical. Co-operation is advocated in the interests of economy, but the accounts of hospitals are difficult to compare, as we have seen, so long as the financial year begins and ends at different dates. The confusion introduced by the existing anomaly will be emphasised when the Government grant is distributed. We would, therefore, point out that no hospital will have a right to complain of neglect or under-assessment so long as the anomaly makes it impossible to compare fairly its position in relation to other hospitals. We invite the Regional Committees of the British Hospitals Association to take this matter in hand at once; and a useful stimulus would be given to the reform if the Voluntary Hospitals Commission would at once give notice that the local committees, before making their grants, must base their comparisons on the accounts for the calendar year from January to December 1920.

